# From commodity to money: The rise of silver coinage around the Ancient Mediterranean (sixth–first centuries bce)

**DOI:** 10.1111/arcm.12615

**Published:** 2020-10-27

**Authors:** F. Albarède, J. Blichert‐Toft, F. de Callataÿ, G. Davis, P. Debernardi, L. Gentelli, H. Gitler, F. Kemmers, S. Klein, C. Malod‐Dognin, J. Milot, P. Télouk, M. Vaxevanopoulos, K. Westner

**Affiliations:** ^1^ Ecole Normale Supérieure de Lyon, CNRS Université de Lyon Lyon France; ^2^ Royal Library of Belgium Brussels Belgium; ^3^ Ecole Pratique des Hautes Etudes Paris France; ^4^ Department of Ancient History Macquarie University Sydney NSW Australia; ^5^ Politecnico di Torino Turin Italy; ^6^ The Israel Museum Jerusalem Israel; ^7^ Institut für Archäologische Wissenschaften Goethe Universität Frankfurt am Main Frankfurt am Main Germany; ^8^ Deutsches Bergbau‐Museum Bochum Germany

**Keywords:** silver coinage, Mediterranean, Pb isotopes, Ag isotopes, monetization

## Abstract

The reasons why the Western Mediterranean, especially Carthage and Rome, resisted monetization relative to the Eastern Mediterranean are still unclear. We address this question by combining lead (Pb) and silver (Ag) isotope abundances in silver coinage from the Aegean, Magna Graecia, Carthage and Roman Republic. The clear relationships observed between ^109^Ag/^107^Ag and ^208^Pb/^206^Pb reflect the mixing of silver ores or silver objects with Pb metal used for cupellation. The combined analysis of Ag and Pb isotopes reveals important information about the technology of smelting. The Greek world extracted Ag and Pb from associated ores, whereas, on the Iberian Peninsula, Carthaginians and Republican‐era Romans applied Phoenician cupellation techniques and added exotic Pb to Pb‐poor Ag ores. Massive Ag recupellation is observed in Rome during the Second Punic War. After defeating the Carthaginians and the Macedonians in the late second century bce, the Romans brought together the efficient, millennium‐old techniques of silver extraction of the Phoenicians, who considered this metal a simple commodity, with the monetization of the economy introduced by the Greeks.

## Introduction

Silver has been highly prized in the Mediterranean and Near East region for millennia. The most obvious traces of its extraction can be seen in the contamination of ice and peat bogs all the way to the Arctic (Hong *et al*. 1994; Cortizas *et al*. 2002). The rise of monetized silver in the late sixth and fifth centuries bce in the Eastern Mediterranean can be interpreted in different ways. Some consider that minting facilitated trade (Kim and Kroll 2008; Davis 2012), and, indeed, the abundance of shipwrecks in the Mediterranean as a proxy for economic performance correlates with Pb peaks observed in the Arctic ice (Parker 1992). In the long run, however, the spread of coinage is also undoubtedly linked to the explosion of Greek mercenary service in the late sixth–fifth centuries, but to an extent that varies with the period and the local political circumstances (Krasilnikoff 1992; De 2019; Trundle 2004). The present study uses Ag and Pb isotopes to further inform the debate by investigating the transition between two modes of silver utilization around the Mediterranean: an early mode in which silver was considered as a simple commodity and traded by weight, and a later monetary mode.

Silver isotopes hold the potential of adding a new dimension to the understanding of early economies first and foremost because silver is a metal of monetary interest and not only another isotopic tracer. As a fingerprinting tool for historical and archaeological applications, Ag isotopes are free from the uncertainties often afflicting Pb isotopes when it comes to untangling silver extraction and recycling (Brill and Shields 1972). Silver and Pb isotope variations reflect processes that are unrelated in nature. Silver isotope variations are due to the minute differences in the thermodynamic properties of the two stable isotopes ^107^Ag and ^109^Ag, known as the ‘isotope effect’ (Fujii and Albarede 2018). The range is very small (on the order of per mil) and essentially nil at the temperatures of magmatic and other high‐temperature (such as cupellation) processes. The latter is true for Pb isotopes as well. In contrast, owing to the radiogenic nature of ^206^Pb, ^207^Pb and ^208^Pb, Pb isotope abundances vary with geological time and tectonic processes. Because Pb isotope ratios change by several per cent upon the decay of U and Th, they vary over a much larger range than the per mil variations created by the isotope effect, which affects Pb isotopes the same way as Ag isotopes, but is drowned in the radiogenic ingrowth not affecting the stable Ag isotopes.

The present work assesses the contribution of combining Ag and Pb isotopic data as a marker of silver extraction and metallurgy in the mining districts which contributed to the monetary mass of the ancient Mediterranean world over time. Much is already known about silver mining techniques and metallurgy (Domergue 2008). Cupellation—the technique of silver purification by smelting in the presence of metallic lead—was known from the Early Bronze Age in the Middle East (Pernicka *et al*. 1998; Pernicka 2004), Attica (Kakavogianni *et al*. 2008) and the island of Thasos (Bassiakos *et al*. 2019). On the Iberian Peninsula, however, evidence for cupellation, often in the form of litharge (Pb oxide), is lacking before the Phoenician colonization in the early first millennium bce (Bartelheim *et al*. 2012; Pérez Macías 2016; Lloréns 2017), which opened up vigorous exchanges between Iberia and the Near East (Neville 2007). From the time of the Second Punic War (240–200 bce) to the second century ce, the Romans took over the silver mining districts of southern Iberia first exploited by the Celtiberians and the Phoenicians from the eighth century bce (Avery 1974; Jurado 2002; Mata 2002), and briefly later on during the mid‐third century bce by the Carthaginians. In the Greek states, the monetary economy was based largely on the exploitation of ores in the Aegean and Thraco‐Macedonia from the second half of the sixth century to the Roman conquest in 148 bce.

A key question for determining the origin of bullion is the nature of ores used as the primary source (galena, sulfides, sulfosalts, chlorides or their alteration products). If Ag and Pb are extracted from the same ore, such as galena, anglesite or cerussite, their isotopes in the purified bullion will reflect the common metal source. If, however, cupellation involves exotic Pb, isotope systematics will instead reflect mixing between the two metal sources. Combined Ag and Pb isotopes can also address whether a particular metal used for coinage was extracted from a primary ore or rather was the product of the remelting of mixed batches of silver fragments, such as silver plates, jewellery or silverware.

**Figure 1:**
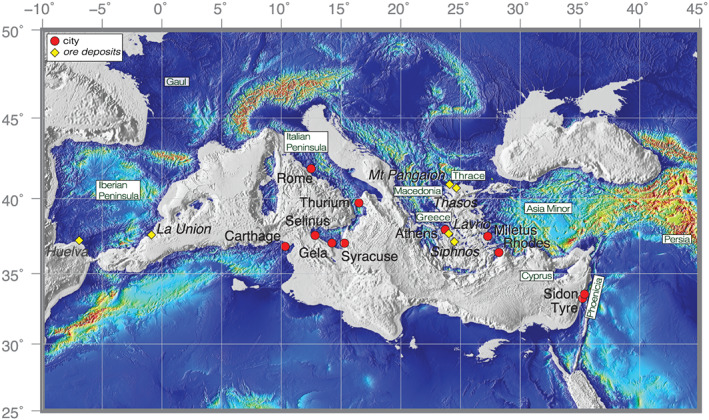
*Map of the Mediterranean with localities (cities and major ore districts) referred to in the text. For clarity, the toponymy used is a mix of names from different periods (e.g., Greece, Asia Minor).* [Colour figure can be viewed at wileyonlinelibrary.com]

Silver isotope variation in coinage (Fujii and Albarede 2018) is much narrower than in ores (Mathur *et al*. 2018; Arribas *et al*. 2020), which potentially allows for a high selectivity of provenance. Correlating minting ages and Pb isotopes has upheld the proposition of a major shift of silver ores shortly after the Second Punic War (Albarède *et al*. 2016; Albarède *et al*. 2020). Here we add to this suggestion by describing correlations between Ag and Pb isotopes on 92 literature and newly acquired data on silver coinage from the Aegean world, Magna Graecia, Carthage and Rome (Fig. 1). With this new expanded study, we reverse Desaulty *et al*.’s (2011) conclusion that Ag and Pb isotopes are uncorrelated and discuss the bearings of the new data set and observed Ag‐Pb correlations on our understanding of silver sources and extraction techniques in the pre‐Roman Empire Mediterranean world.

**Figure 2:**
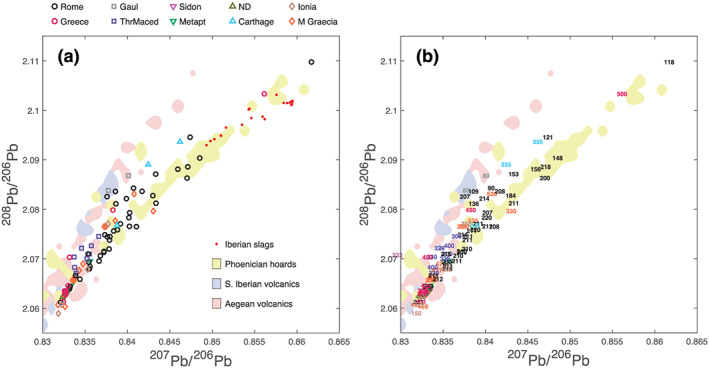
*Lead isotope relationships for the samples analysed here and literature data: the graphs show the same samples with symbols (a) and minting ages (bce) (b), respectively. In spite of substantial overlap, (b) is meant to reduce the number of different symbols and improve legibility, while helping with the age of minting. The fields representing recent Aegean and south Iberian volcanic rocks (downloaded from the GEOROC database;* http://georoc.mpch‐mainz.gwdg.de/georoc/ *) are clearly relevant to Aegean and Magna Graecia coinage (primary ores). The points to the right attest to the addition of exotic Pb to Ag ores during cupellation. The overlap between these points, the Phoenician hoards from the 10th–9th centuries bce, and the Ibero‐Punic slags and silver objects found in southern Iberia (Anguilano* et al. *2010* *; Murillo‐Barroso* et al. *2016* *) show that first Carthage, then Rome in the late second century bce, had adopted the Phoenician smelting process.* [Colour figure can be viewed at wileyonlinelibrary.com]

The present study combines literature data with 14 new Pb isotope data and 42 new Ag isotope data (see Table S1 in the additional supporting information). The Pb isotope compositions are reported in Figure 2 with symbols for proper identification and dates for minting age (the median of the uncertainty range). The fields of volcanic rocks (Albarède *et al*. 2020; http://georoc.mpch‐mainz.gwdg.de/georoc/) genetically related to magmatic hydrothermal ore deposits (light blue for southern Iberia, pink for the Aegean) from the literature are also plotted to show how closely the main group comprising most samples from Thrace, Greece and the Greek colonies (Magna Graecia) track potential lead sources. Also plotted is the field of Pb isotopes in the Near Eastern silver hoards of *hacksilber* (chopped silver plates and jewellery) from Dor, Akko and Ein Hofez (10th–9th centuries bce), which are contemporaneous with the first Phoenician settlements around the Mediterranean, especially in southern Iberia (Neville 2007; Eshel *et al*. 2019). Particularly striking is a major difference in the apparent source of the metal used to mint the *denarii* from the Roman Republic (Albarède *et al*. 2020; Westner *et al*. 2020). The majority of late *denarii* (in black) plot to the right of the coins struck before the Second Punic War to slightly after and they conspicuously overlap the fields of Phoenician hoards (Eshel *et al*. 2019) as well as the field of the Phoenician and Ibero‐Punic slags and silver objects found in southern Iberia (Anguilano *et al*. 2010; Murillo‐Barroso *et al*. 2016). Although more data clearly are needed, Pb isotopes from three Carthaginian and two Gaulish coins also plot to the right, but at higher ^208^Pb/^206^Pb values.

**Figure 3:**
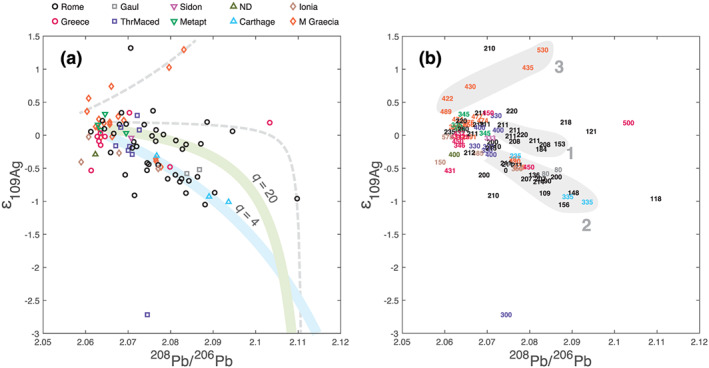
*Correlation between* ^*109*^ *Ag/* ^*107*^ *Ag, expressed using the epsilon (*ε*) notation (deviation from a reference ratio in parts per 10 000), and* ^*208*^ *Pb/* ^*206*^ *Pb for 92 silver coins from the Aegean world, Magna Graecia, Carthage and Rome. In spite of substantial overlap, (b) is meant to reduce the number of different symbols and improve legibility, while helping with the age of minting. Groups are referred to with light grey roman numerals. The same data are represented with symbols (a) and minting ages bce (b). (a) Mixing lines do not display as linear arrays but as hyperbolae (see the additional supporting information). The curves are shown as references and were not obtained by a fit to the data. The curvature is a function of the ratio (Pb/Ag)*_*ore*_ *in the ore (or recycled bullion) and (Pb/Ag)*_*Pb*_ *in the Pb used for smelting and* q *is the ratio (Pb/Ag)*_*ore*_ */(Pb/Ag)*_*Pb*_ *. For Group 1 (green mixing hyperbola), (Pb/Ag)*_*ore*_ *= 20 (Pb/Ag)*_*Pb*_ *, which points to a Ag‐rich silver source. In contrast, for Group 2 (blue mixing hyperbola), (Pb/Ag)*_*ore*_ *= 4 (Pb/Ag)*_*Pb*_ *, which suggests a Ag‐poor source. Group 1, consisting of coins minted around the Second Punic War (see b), clearly represents remelting of silver bullion, jewellery or debased coinage. Group 2, comprising coins minted by Carthage, some Magna Graecia colonies and the late Roman Republic, represents coins minted from Iberian ores after cupellation with exotic Pb. In contrast, coins pertaining to the largest group of Greek and Magna Graecia ores and plotting near the apex of this bundle was cupellated using Pb cogenetic with silver ores. Five Magna Graecia coins (Group 3) definitely have been cupellated with Pb containing Ag with positive* ε_*109Ag*_ *values. The dashed grey hyperbolae show more potential mixing arrays: shallow slopes, both positive and negative, indicate probable multiple events of cupellation.* [Colour figure can be viewed at wileyonlinelibrary.com]

**Figure 4:**
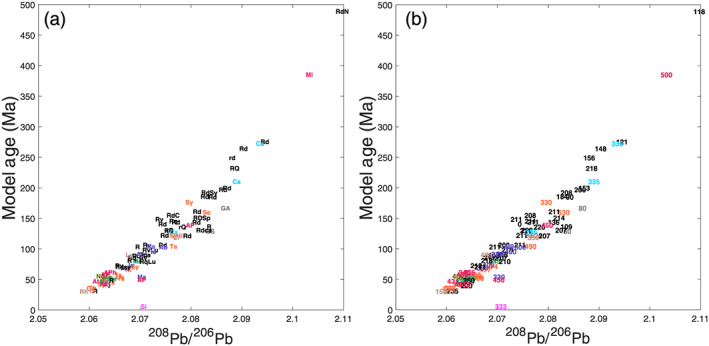
*Correlation between model ages (millions of years) as calculated by Albarède* et al. *(2012) and* ^*208*^ *Pb/* ^*206*^ *Pb ratios: (a) for abbreviations used as symbols, see the additional supporting information under the header ‘ID’ (Table* *S1* *); and (b) minting ages bce.* [Colour figure can be viewed at wileyonlinelibrary.com]

Silver isotope compositions are reported as *ε*_109Ag_ values (the deviation in parts per 10 000 of the ^109^Ag/^107^Ag ratio of a given sample from that of the Ag standard SRM NIST 978a) (Desaulty *et al*. 2011). All the data fall into four well‐defined groups. We found that the clearest representation is *ε*_109Ag_ versus ^208^Pb/^206^Pb (Fig. 3). Model ages (Albarède et al., 2012) could have been used as well, but since ^208^Pb/^206^Pb ratios correlate strongly with model ages (Fig. 4), we opted for the former, which is more familiar to the community of archaeometry.

In the *ε*_109Ag_ versus ^208^Pb/^206^Pb plot, the data form a bundle of arrays radiating from *ε*_109Ag_ ~ 0 and ^208^Pb/^206^Pb = ~2.065:

- Group 1trends slightly to the right of *ε*_109Ag_ ~ −0.25.
- The well‐populated Group 2 trends more steeply towards *ε*_109Ag_ ~ −1.0.
- Group 3 trends towards positive values.

These groups are well delineated at high ^208^Pb/^206^Pb. A few samples cannot be attributed to one group or another, while some rogue points are arguably difficult to explain, such as the low *ε*_109Ag_ Abydos drachma. With these restrictions in mind, most data points of Group 1 are Roman Republic *denarii* minted before the Second Punic War to slightly after. Most data points of Group 2 are coinage from Carthage and Roman Republic *denarii* minted after the Second Punic War. The two Gaulish samples belong to this group. Five coins from Group 3 were minted in southern Italy and Sicily (Thurium, Gela, Syracuse and Selinus). Most coins from classic Greece and Magna Graecia plot at the apex of the bundle (^208^Pb/^206^Pb ~ 2.065) and hence can be considered as pertaining to either all or none of the groups, in which latter case they can be considered to define a fourth group.

The coins from Groups 1–3 plot within a narrow range of ±1 *ε*_109Ag_. This range is conspicuously smaller (Fujii and Albarede 2018) than the spread of silver ore deposits (Mathur *et al*. 2018; Arribas *et al*. 2020). Group 2 shows a well‐defined negative correlation between *ε*_109Ag_ and ^208^Pb/^206^Pb. This group, with few exceptions, corresponds to the field to the right of the magmatic trend in Figure 2. Given that Ag and Pb isotope fractionation is due to processes physically independent of each other, this array attests to mixing between two end‐members, for example silver ore and lead metal used for cupellation. The idea that lead in silver coins comes from a mixture of lead and silver ores is not new (Brill and Shields 1972; Anguilano *et al*. 2010; Eshel *et al*. 2019), but Pb isotopes by themselves do not suffice to substantiate mixing, first because ores often form from mixed sources, and second because lead is a metallurgical additive to the silver ore indispensable for silver purification.

**Figure 5:**
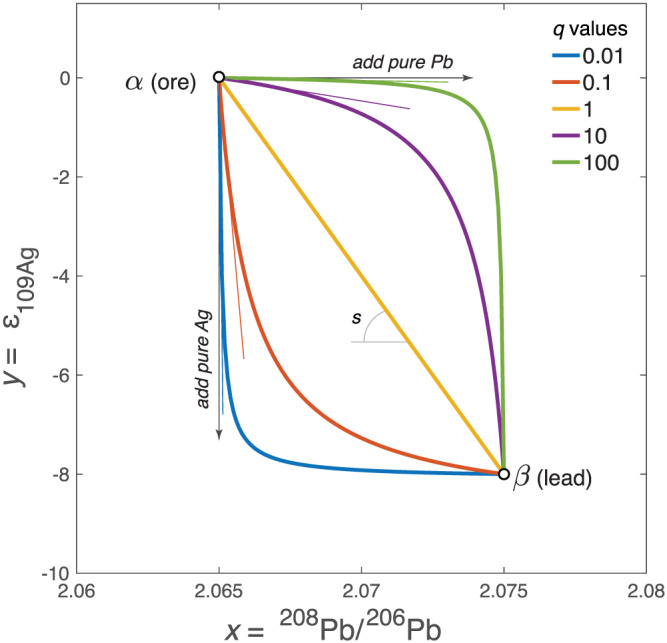
*Hyperbolae representing mixing between a Ag‐rich ore* α *and metallic Pb* β *used for smelting for different values of* q *= (Pb/Ag)* ^β^ */(Pb/Ag)* ^α^ *. The thin coloured lines represent tangents to the corresponding mixing hyperbolae at* x *=* x^α^ *and* y *=* y^α^ *(see the text).* s = (y^β^ − y^α^)/(x^β^ − x^α^) *.* [Colour figure can be viewed at wileyonlinelibrary.com]

At this stage, it is essential to emphasize the non‐linear property of mixing in ratio–ratio plots when the denominators, here ^107^Ag and ^206^Pb, are different (see Appendix A). This can be simply cast by reasoning that adding pure Pb to a mixed silver–lead ore leaves Ag with the *ε*_109Ag_ signature of the ore. In contrast, mixing changes ^208^Pb/^206^Pb and the other isotopic ratios, but only if Pb from the ore and the cupellation metal have different origins. The curvature of mixing hyperbolae increases with the contrast of ^107^Ag/^206^Pb, or accepting, for sake of simplification, a negligible inaccuracy, the Ag/Pb ratios between the two end‐members. Reference hyperbolae going through two geologically and historically acceptable end‐members have been calculated and drawn in Figure 4 using the simple and useful relationship derived in Appendix A. In the *ε*_109Ag_ versus ^208^Pb/^206^Pb plot, the slope of each array at the Ag‐rich end‐member (Fig. 5) is a particularly useful parameter. When comparing two groups, say Groups 1 and 2, the Pb/Ag ratio of the Ag‐rich group relates to the corresponding slope through:

(1) $$\frac{{\left(\mathrm{Pb}/\mathrm{Ag}\right)}_{2}}{{\left(\mathrm{Pb}/\mathrm{Ag}\right)}_{1}}=\frac{{\text{slope}}_{1}}{{\text{slope}}_{2}}$$

Note that the hyperbolae drawn in Figure 4 were not fitted through the actual data but were drawn for reference only. Silver ore smelted for the peri‐Punic Group 1 is clearly much richer in Ag than that used for Group 2. Mixing within Group 2 (in blue) corresponds to a Pb/Ag ratio in the silver ore only a factor of approximately four times lower than in the Pb metal used for cupellation. The ore was therefore fairly poor in Ag. In contrast, the silver ore of Group 1 (in green) has a Pb/Ag ratio 20 times smaller than in the Pb additive. Combining Ag and Pb isotope analysis of Roman coins confirms evidence from archaeological excavations (Anguilano *et al*. 2010; Murillo‐Barroso *et al*. 2016; Eshel *et al*. 2019) that the two metals came from distinct sources and had to be combined for efficient Ag extraction. The range in Pb isotope compositions of Roman ingots from Iberia is rather narrow (Trincherini *et al*. 2009), while the *ε*_109Ag_ values of Iberian galenas, although more variable, are negative (Arribas *et al*. 2020).

Most of the data in Figure 4 cluster around *ε*_109Ag_ ~ −0.25 and ^208^Pb/^206^Pb = 2.06–2.08. The ^207^Pb/^206^Pb range is ambiguous. But, as pointed out by Fujii and Albarède (2018), the narrow range of *ε*_109Ag_ (−1 to 2 per 10 000) in silver coinage, whether minted around the Mediterranean during antiquity, in medieval or pre‐modern Europe, or in the Spanish Americas, contrasts markedly with the broad scatter of several per mil observed for potential silver ores (Arribas *et al*. 2020). It is remarkable that the mean *ε*_109Ag_ in silver coinage differs from the (rare) mantle values, but is indistinguishable from the value of the organic‐rich Cody Shale (Theis *et al*. 2013) deposited some 85 Ma ago. This observation is consistent with the suggestion that the metals of Pb‐Zn(‐Ag) ores were eventually derived not from the mantle, but from oceanic black shales deposited in anoxic environments (Milot et al. submitted).

The range of *ε*_109Ag_ values among the Ag‐rich end‐members is very narrow, within ±0.2 epsilon units of the SRM NIST 978a value. Such striking lack of fractionation of Ag isotopes suggests that the source ores formed primarily in a high‐temperature environment, usually qualified as mesothermal (200–400°C) (Guilbert and Park 2007). It shows that the silver ores, presumably sulfides, did not go through intense weathering or any other low‐temperature process that could have readily fractionated Ag isotopes (Evans 2009). The ores used as Pb sources could be galena and cerussite, as is the case for the Athenian Lavrion district (Conophagos 1980) and southern Iberia (Baron *et al*. 2017). The contrast between the Aegean and non‐Aegean coins implies that the source of Ag in the former went through cupellation using geologically young (Cenozoic), and probably cogenetic, Pb and Ag. Extraction of the two metals in the Aegean must have taken place at a short distance from each other.

The new perspective provided by Ag isotopes and derived from the curvature of Ag‐Pb mixing hyperbolae allows the pursuing of the suggestion based on archeometallurgical observations (Rowan 2013), statistics on triumph and temple treasure composition (de Callataÿ 2006), and Pb isotopes on silver coinage (Birch *et al*. 2020b) that the Western Mediterranean used Greek coinage as a source of bullion. Silver‐lead isotopic correlations show that Ag from Group 1, most likely delivered by Carthage after the First Punic War, was reprocessed by the Romans. In contrast, the two Gaulish coins belong to Group 2 and, therefore, were not recupellated from pre‐existing bullion.

Silver ores smelted for Group 3 (Gela, Thurium, Selinus, Syracuse and cities from Magna Graecia with a long history of war and shared interests) were cupellated with unusual lead ores containing Ag with positive *ε*_109Ag_ values, but unfortunately of so‐far unknown origin.

The corollary of these observations is:

- Silver from most Aegean and Magna Graecia samples was smelted with lead from local ores. Having both silver and lead sources at one's disposal locally, possibly from the same mining district, perhaps the Lavrion, seems to have been key to secure silver production.
- In contrast, silver mined by Carthage in the fourth century bce and by the Romans in the second half of the second century bce was cupellated with geologically older lead extracted at some distance from the silver ores.
- Metal used for Roman coins at the time of the Second Punic War was obtained from recalled silver (Albarède *et al*. 2016) and purified with lead unrelated to any identifiable silver mining districts.

Silver used for minting Roman coinage during the Second Punic War (Group 1) was minted only during a short period consistent with a discontinuity in the Ag isotopic composition noted by Albarède *et al*. (2016) at the time of the Roman monetary reform of 213 bce (this date is used here for convenience; the exact date is still a matter of debate though not too far from 213 bce). This change in silver source most likely corresponds to domestic remelting of massive amounts of silver metal from the war penalties paid by Carthage after the First Punic War and the Mercenary wars. Given the apparent success of the silver recall in Rome at the climax of the Second Punic War (Livy, XXIII, 48.9), Carthaginian silver must have been partly in private hands, mostly as silverware and jewellery, and partly in the form of debased *quadrigati*. Cupellation using scrap lead of non‐descript origin, possibly from Sardinia, which Rome annexed in 238 bce, must have followed in the wake of the unsuccessful debasement attempted during the monetary reform of 213 bce.

The narrow range of Pb/Ag ratios displayed by each group requires explanation. The answer lies in the metallurgical process. Silver lost to the litharge by oxidation steeply increases with metal fineness (Swinbourne *et al*. 2003; Ueda *et al*. 2005). In other words, making very pure silver comes at the price of wasting more of it. The metallurgist therefore has to play with two parameters: oxygen flow and the Pb/Ag ratio of the silver ore–lead metal mixture to find the optimum ore–lead mixture that will provide the purest silver without losing too much of it to the slag (see Appendix B). Two‐step cupellation as proposed for Lavrion (Pernicka and Bachmann 1983) would manifest itself by sharply curved mixing hyperbolae unless silver and lead ores are very similar in both components. Archaeological finds from many Phoenician settlements attest that the art of cupellation applied to low‐grade ores, typically jarosite earth from Rio Tinto in the lower Guadalquivir Valley near the modern town of Huelva, Spain, using metallic lead had been mastered to the highest degree well before the first Lydian (late seventh century bce) and Aegean (late sixth century bce) coinages were struck (Blanco and Luzón 1969). The Pb concentrations in coins from the Roman Republic (Hollstein 2000; Westner *et al*. 2020) fall within a narrow range and are systematically lower than the solubility of Pb in Ag (1–3 wt% between 300 and 900°C; Karakaya and Thompson 1987). This observation together with the low vapour pressure of Pb, leaves no doubt that Pb was not added to the metal after purification.

Once the Carthaginians were defeated at Zama in 202 bce, it took the Romans several decades to control the land of the Celtiberian tribes (Appian, 6; Richardson and Richardson 2004) and organize mining and silver extraction. The present data provide evidence that the Romans only recommenced large‐scale exploitation of jarositic silver ores of the Iberian Pyrite Belt and Rio Tinto (Domergue 1990; Westner *et al*. 2020) in the late second century bce. They clearly did not adopt the Greek method of local mining of high‐grade ores, but used the Phoenician protocol, also mastered by the Celtiberian tribes, of combining low‐grade silver ores with galena‐sourced Pb from the entire Iberian Peninsula.

The Eastern and Western Mediterranean civilizations looked at the same metal, but from different perspectives. Phoenicia (Gitler and Tal 2006; Elayi *et al*. 2009) and Carthage (Jenkins 1974), although well aware of coined silver in the Aegean world, primarily considered this metal as a precious commodity, just like ivory, myrrh and amber. Only with the arrival of Greek mercenaries in the second half of the fifth century bce did these authorities adopt coinage. Not all the Greeks, however, struck silver as intensively as did Athens and a few other city‐states later on and, later on again, Hellenistic kingdoms. The Western Mediterranean, especially Carthage and Rome, also resisted monetization for longer than did the Greek world. From the inventories of Roman triumphs and temples in the second century bce, de Callataÿ (2006) concluded that at least 80 wt% of the available silver was left as bullion (*infecti*). Monetized silver does not seem to have become prevalent relative to jewellery and bullion, as attested to by persistent references to large sums in weight units (37). The overall coincidence between the isotopic differences and the historical gap between Groups 1 and 2 testifies to the reluctance of the Roman Republic to use coined money for larger transactions. Indemnities inflicted on the defeated Carthaginians reported by Polybius in his *Histories* and on Macedonians reported by Livy (XXXIII, 30.7), always labelled in talents and not in any sort of monetary denomination, attest to the Romans being uneasy about pervasive monetization until reliable sources of bullion were safely established. Numismatic evidence supporting the rapid expansion of silver coinage in the second half of the second century bce and its potential causes, such as pressure on the copper supply for minting (Bransbourg 2017), are reviewed in detail by Kay (2014).

## Materials and methods

Silver isotopes were analysed on series of coins from the Roman Republic (Westner *et al*. 2020) and Magna Graecia (Birch *et al*. 2020a, 2020b). Drill cores previously analysed for Pb isotopes were reused for Ag isotopes. Drill cores of Punic War *denarii* from Spain (provided by Pierluigi Debernardi) and of 10 drachmas from different periods of Thrace, Athens, Miletus, Rhodes, Cilicia and Gela (Birch *et al*. 2020a) were analysed for both Pb and Ag isotopes. Literature data (Desaulty *et al*. 2011; Albarède *et al*. 2016) complemented the data set. Analytical techniques have been described elsewhere (Desaulty *et al*. 2011).

### PEER REVIEW

The peer review history for this article is available at https://publons.com/publon/10.1111/arcm.12615.

## Supporting information

Table S1. Multi‐collector inductively coupled plasma mass spectrometer (MC‐ICP‐MS). OK as edited? Pb and Ag isotopic data from the present work and the literature.

## Appendix A Mixing equation for ratios

The mass balance of isotope ratios *x* and *y* upon mixing of end‐members *α* and *β* requests (Albarède 1995):

(A1) $$\begin{array}{c} x=\varphi_{\mathrm{Pb}}^{\alpha }x^{\alpha }+\varphi_{\mathrm{Pb}}^{\beta }x^{\beta }\\ y=\varphi_{\mathrm{Ag}}^{\alpha }y^{\alpha }+\varphi_{\mathrm{Ag}}^{\beta }y^{\beta } \end{array}\;\text{with}\;\begin{array}{c} \varphi_{\mathrm{Pb}}^{\alpha }+\varphi_{\mathrm{Pb}}^{\beta }=1\\ \varphi_{\mathrm{Ag}}^{\alpha }+\varphi_{\mathrm{Ag}}^{\beta }=1 \end{array}$$

where

(A2) $$\varphi_{\mathrm{Pb}}^{\beta }=\frac{f^{\beta }C_{\mathrm{Pb}}^{\beta }}{f^{\alpha }C_{\mathrm{Pb}}^{\alpha }+f^{\beta }C_{\mathrm{Pb}}^{\beta }}\;\text{with}\;f^{\alpha }+f^{\beta }=1$$

is the mass fraction of Pb contributed by end‐member *β*, where *f* ^*α*^ and *f* ^*β*^ are the weight fraction of end‐members *α* and *β*, respectively; and *C* is the weight concentration of the species at the denominator (^206^Pb for *x* and ^107^Ag for *y*). Similar definitions apply to the other variables. Replacing $\varphi_{\mathrm{Pb}}^{\alpha }$ by $1-\varphi_{\mathrm{Pb}}^{\beta }$, we obtain

(A3) $$x-x^{\alpha }=\varphi_{\mathrm{Pb}}^{\beta }\left(x^{\beta }-x^{\alpha }\right)$$

or

(A4) $$\frac{x^{\beta }-x^{\alpha }}{x-x^{\alpha }}=\frac{1}{\varphi_{\mathrm{Pb}}^{\beta }}=1+\frac{f^{\alpha }C_{\mathrm{Pb}}^{\alpha }}{f^{\beta }C_{\mathrm{Pb}}^{\beta }}$$

Equation (A4) can be rearranged, with a similar equation for *y* as

(A5) $$\frac{x^{\beta }-x}{x-x^{\alpha }}=\frac{f^{\alpha }C_{\mathrm{Pb}}^{\alpha }}{f^{\beta }C_{\mathrm{Pb}}^{\beta }}\;\text{and}\;\frac{y^{\beta }-y}{y-y^{\alpha }}=\frac{f^{\alpha }C_{\mathrm{Ag}}^{\alpha }}{f^{\beta }C_{\mathrm{Ag}}^{\beta }}\;$$

Dividing the equation for *y* by the equation for *x* gives

(A6) $$\frac{y^{\beta }-y}{y-y^{\alpha }}=\frac{{\left(\mathrm{Pb}/\mathrm{Ag}\right)}^{\beta }}{{\left(\mathrm{Pb}/\mathrm{Ag}\right)}^{a}}\times \frac{x^{\beta }-x}{x-x^{\alpha }}=q\frac{x^{\beta }-x}{x-x^{\alpha }}$$

At the expense of a negligible error, ^206^Pb/^107^Ag for *y* ratios have, for simplicity, been replaced by the elemental weight ratios Pb/Ag. Expressing the isotopic ratios, we finally obtain:

(A7) εAg109β−εAg109εAg109−εAg109α=Pb/AgβPb/Aga×Pb/Pb/206208β−Pb/Pb206208Pb/Pb206208−Pb/Pb206208α

Equation (A7) is represented by a hyperbola when the Pb/Ag ratios of the two end‐members are different and by a straight line when they are identical (Fig. 5). An alternative equation can be derived:

(A8) $$\left(x-x_{0}\right)\left(y-y_{0}\right)=x_{0}y_{0}-\gamma$$

where

(A9) $$x_{0}=\frac{x^{\alpha }-qx^{\beta }}{1-q},y_{0}=\frac{y^{\beta }-qy^{\alpha }}{1-q},\gamma =\frac{x^{\alpha }y^{\beta }-qx^{\beta }y^{\alpha }}{1-q}$$

where *q* is inferred from the slope of the mixing hyperbola (d*y*/d*x*)_*α*_ at *x* = *x* ^*α*^. After tedious, but straightforward, manipulations, the relationship

(A10) $$q=s/{\left(dy/dx\right)}_{\alpha }$$

is obtained, where *s* = (*y*^*β*^ − *y*^*α*^)/(*x*^*β*^ − *x*^*α*^).

A useful expression relating *q*, *φ*_Pb_, and *φ*_Ag_ can also be derived:

(A11) $$\varphi_{\mathrm{Ag}}^{\beta }=\frac{\varphi_{\mathrm{Pb}}^{\beta }}{\varphi_{\mathrm{Pb}}^{\beta }+q\left(1-\varphi_{\mathrm{Pb}}^{\beta }\right)}$$

## Appendix B Optimum Ag/Pb for smelting

To obtain a good approximation, the weight fraction *f*_slag_ of slag obtained by cupellating the ore–lead mixture is equal to the concentration

(B1) $$C_{0}^{\mathrm{Pb}}=\frac{\left[\mathrm{Pb}\right]}{\left[\mathrm{Pb}\right]+\left[\mathrm{Ag}\right]}$$

in the mixture, where brackets stand for concentrations. The fraction of total silver lost to the slag is approximately

(B2) $$f_{\text{slag}}K_{\text{slag}/\mathrm{met}}^{Ag}\approx C_{0}^{\mathrm{Pb}}K_{\text{slag}/\mathrm{met}}^{\mathrm{Ag}}$$

where $K_{\text{slag}/\mathrm{met}}^{Ag}\;$ is the coefficient of the Ag fractionation between the slag and purified Ag. Reducing the amount of Pb in the original ore–Pb mixture both reduces $C_{0}^{\mathrm{Pb}}$ and increases $K_{\text{slag}/\mathrm{met}}^{\mathrm{Ag}}$ (Ueda *et al*. 2005), which requires the proportions to be optimized before extraction. This result justifies that the Pb/Ag ratio during cupellation is constrained within a relatively narrow range.
